# Supplementary material for: Frequency of monocyte subsets is linked to the severity of atherosclerosis in patients with ischemic heart disease: A case-control study
Source: Biomedicine (Taipei). 2020 Jun 5;10(2):36–47. doi: 10.37796/2211-8039.1015 (PMC7608850; doi:10.37796/2211-8039.1015)
Supplement: Supplementary file 1 [file bmed-10-02-036-s001.pdf]

BioMedicine

Manuscript 1015

Frequency of monocyte subsets is linked to the severity of atherosclerosis in patients with ischemic heart disease: a case-control study

Irina V. Kologrivova

Tatiana Suslova

Olga Koshelskaya

Oksana Trubacheva

Olga Haritonova

See next page for additional authors

Follow this and additional works at: https://www.biomedicinej.com/biomedicine

Part of the Cardiovascular Diseases Commons, Diagnosis Commons, Life Sciences Commons, and the Medical Immunology Commons

This work is licensed under a Creative Commons Attribution 4.0 License.

Frequency of monocyte subsets is linked to the severity of atherosclerosis in patients with ischemic heart disease: a case-control study

Authors

Irina V. Kologrivova, Tatiana Suslova, Olga Koshelskaya, Oksana Trubacheva, Olga Haritonova, and Irina Vinnitskaya

ORIGINAL ARTICLE

Frequency of monocyte subsets is linked to the severity of atherosclerosis in patients with ischemic heart disease: A case-control study

Q6 Irina V. Kologrivova*, Tatiana Suslova, Olga Koshelskaya, Oksana Trubacheva, Olga Haritonova, Irina Vinnitskaya

1. Background

Atherosclerosis is an underlying cause of the development of acute cardiovascular events, the most dangerous of which are myocardial infarction and stroke, characterized by high morbidity and mortality all over the world [1]. Nowadays, it is widely accepted that atherosclerosis is associated with the development of inflammation [2]. Monocytes are recognized as central cells in the development of inflammation process. Their accumulation in response to injury of the endothelium followed by the subsequent differentiation into the macrophages and further transformation into the foam cells is one of the primary events in the course of atherogenesis [3, 4].

In rodent models, the monocytes with high expression of surface marker Ly6C (Ly6C^{hi} monocytes) predominate in the circulation and are termed “classical” monocytes, whereas the monocytes with low expression of this marker (Ly6C^{lo}) are known as “non-classical” monocytes [5]. Ly6C marker is absent in human monocytes. However, an alternative approach was undertaken to categorize human monocytes into subpopulations based on differential expression of CD14 and CD16 molecules. Human classical monocytes have phenotype CD14++CD16^{lo}, while non-classical are CD14+CD16^{hi} (abbreviations such as CD14+CD16- and CD14^{dim}CD16+ may be used as an equivalent). Intermediate CD14++CD16^{hi} monocytes are also distinguished [6].

Subpopulations of monocytes are not absolutely discrete and are characterized by high plasticity. After a release from the bone marrow, the classical monocytes may further differentiate into the non-classical monocytes acquiring intermediate phenotype during this process. Differentiation of monocytes from classical to non-classical has been shown to be dependent upon the nuclear receptor Nr4a1 (Nur77) [7].

There exists a considerable discrepancy when it comes to definition of the functions of monocyte subpopulations and their input in the progression of atherosclerosis. Numerous works demonstrated that CD16^{hi} monocytes (both non-classical and intermediate) are characterized by the inflammatory phenotype. Indeed, they more abundantly express surface marker CD86, TNF receptor (TNFR)-2, and Toll-like receptor (TLR)-2, and produce higher levels of inflammatory cytokines upon stimulation compared to classical monocytes, which, on the contrary, express high levels of surface markers CD163 and CD93 [8, 9]. Some authors showed that classical monocytes from normolipidemic mice mediate plaque regression upon recruitment and differentiation to the anti-inflammatory M2 macrophages [10]. On the other hand, many works suggest that classical CD14++CD16^{lo} monocytes represent an inflammatory subpopulation with a causative role in atherogenesis, whereas non-classical monocytes are considered to fulfill mainly patrolling function in the vascular wall [5, 6, 11, 12].

Understanding the mechanisms, which determine monocyte-dependent effects in atherosclerosis, may provide the new diagnostic and therapeutic tools. This provides a rationale for an in-depth study of the patterns of monocyte subpopulations in atherosclerosis.

2. Aim

The present study aimed to assess the relationships between the different subpopulations of monocytes and metabolic and inflammatory factors

Q7 Received 7 May 2020; accepted 7 May 2020.
Available online ■ ■ ■

* Corresponding author at: 634012, Kievskaya 111a, Tomsk, Russia.
E-mail address: ikologrivova@gmail.com (I.V. Kologrivova).

https://doi.org/10.37796/2211-8039.1015
2211-8020/Published by China Medical University 2020. © the Author(s). This is an open access article under the CC BY license (http://creativecommons.org/licenses/by/4.0/).

that may affect monocyte functioning and atherosclerosis severity in patients with stable coronary heart disease.

3. Methods

3.1. Patients

The study protocol was approved by the local Biomedical Ethics Committee of Cardiology Research Institute (protocol #139 from November 18, 2015) as part of the fundamental interdepartmental theme #0548-2014-0018, #30 titled "Fundamental aspects of onset and development of socially significant cardio-vascular diseases, revealing of the targets for improvement of diagnostics, treatment, and prognosis and the mechanisms of protection". All patients gave the informed consent to participate in the study. All procedures were performed in accordance with the Helsinki Declaration and principles of Good Clinical Practice (GCP) and Good Laboratory Practice (GLP).

A total of 26 patients, both men and women with stable ischemic heart disease (IHD) undergoing coronary angiography (CA), including four patients without coronary stenosis with myocardial ischemia verified by the cardiac stress tests (stress echocardiography or myocardial scintigraphy) were recruited in the study from the Department of Atherosclerosis and Coronary Artery Disease of Cardiology Research Institute, Tomsk NRMC. Among all the recruited patients, 17 patients had significant coronary artery disease defined as diameter stenosis of more than 70%. Coronary angiography was performed in all patients with imaging system INNOVA (GE Medical Systems, USA). Severity of CHD was assessed by the Gensini score (GS). For this purpose, eight segments of the coronary arteries were identified as follows: the left coronary artery trunk, the anterior descending artery, the first diagonal artery, the second diagonal artery, the circumflex artery, the marginal artery, the right coronary artery, and the posterior descending artery. Degree of stenosis and localization of the stenotic changes were determined with assignment of the appropriate coefficient. Patient characteristics are presented in Table 1.

In addition, six patients without IHD were recruited in the study to assess the frequencies of monocytes subpopulations and lipid profiles.

Patients were excluded from the study if any of the following was present: acute coronary event such as transitory ischemic attack, acute coronary syndrome, and acute myocardial infarction within 6 months before the study; coronary artery bypass grafting within6 months before the study; obesity class II and higher (body mass index (BMI) > 35); confirmed symptomatic forms of arterial hypertension; severe comorbidity (hepatic failure, kidney failure, and oncological diseases); the presence of gastrointestinal pathology; and refusal to participate in the study.

All patients recruited in the study received standard antihypertensive therapy including combination of renin-angiotensin-aldosterone system blockers (78%), indapamide retard (24%), calcium channel blockers (52%), and beta-blockers (80%). The majority of recruited patients (81%) received

Table 1. Characteristics of patients with coronary artery disease depending on the percentage of coronary artery stenosis (Me (Q1; Q3)).

Parameter	Patients with coronary stenosis <70% (n = 9)	Patients with coronary stenosis ≥70% (n = 17)	p
Men/women	4/5	8/9	0.613
Age, years	65.0 (60.0; 66.0)	64.0 (56.0; 65.0)	0.525
GS	5.0 (0; 9.5)	42.0 (20.0; 72.0)	**<0.001** Q4
Systolic blood pressure, mm Hg	120.0 (120.0; 130.0)	125.0 (117.5; 134.5)	**0.045**
Diastolic blood pressure, mm Hg	70.0 (70.0; 78.0)	77.0 (64.5; 80.0)	0.121
AH duration, years	5.0 (3.0; 15.0)	16.0 (12.0; 30.0)	**<0.001**
DM presence	2 (22%)	9 (52.9%)	0.217
DM duration, years	0 (0; 0)	3.0 (0.0; 14.0)	**0.047**
Smoking	3 (33.3%)	6 (35.3%)	1.000
Smoking duration, years	11.5 (3.0; 20.0)	33.0 (30.0; 40.0)	0.071
Body mass index, kg/m^2	28.6 (28.0; 31.0)	30.3 (29.0; 31.6)	**0.028**
Waist circumference, cm	98.0 (96.0; 101.0)	102.5 (93.0; 106.0)	**0.043**
Visceral adiposity index	1.7 (1.4; 4.7)	2.9 (2.2; 3.4)	0.052
Fasting glucose, mM	5.2 (4.9; 5.8)	5.9 (5.0; 6.8)	**0.015**
Fasting insulin, μIU/mL	8.7 (7.5; 9.5)	6.5 (5.2; 11.2)	0.388
HOMA	1.9 (1.7; 2.3)	2.0 (1.6; 6.6)	0.113
hsCRP, mg/L	2.1 (1.0; 3.3)	3.7 (1.8; 7.6)	**0.040**

GS – Gensini score; AH – arterial hypertension; DM – diabetes mellitus; HOMA – homeostatic model assessment for insulin resistance; hsCRP – high-sensitive C-reactive protein.

low doses of statins (atorvastatin at a mean dose of 15 mg and rosuvastatin at a mean dose of 7.5 mg). Diabetes mellitus type 2 (DM2) patients received oral glucose-lowering medications, including biguanides (100% of DM2 patients), sulphonylurea derivatives (50% of DM2 patients), and inhibitors of dipeptidyl peptidase 4 (30% of DM2 patients).

Visceral adiposity index (VAI) was calculated using the following formulas: VAI = (WC/(39.68 + (1.88 × BMI))) x (TG/1.03) x (1.31/HDL) in male patients and VAI = (WC/(36.58 + (1.89 × BMI))) x (TG/0.81) x (1.52/HDL) in female patients: where WC and BMI are waist circumference and body mass index, respectively [13].

Fasting samples of peripheral blood were obtained into 4-mL heparinized tubes, 4-mL tubes with EDTA, and 10-mL tubes without anticoagulant.

3.2. Biochemical analysis

Insulin concentrations were evaluated in serum by enzyme-linked immunosorbent assay with AccuBind kits (Diagnostic System Laboratories, USA). Serum concentration of glucose was measured by hexokinase method using analyzer BIOSEN C-line Clinic (EKF diagnostic, Germany). Enzyme colorimetric method was used to estimate serum concentration of total cholesterol, triacylglycerol, and high-density lipoprotein (HDL) cholesterol (Diakon, Russia). Concentration of low-density lipoprotein (LDL) cholesterol was calculated as [LDL] = [Total cholesterol] − [Triacylglycerol (TG)] − [HDL]. Atherogenic index was calculated as [Total cholesterol] − [HDL])/[HDL]. Glycated hemoglobin (HbA1c) content was measured by immunoturbidimetric method (DiaSys, Germany). Homeostatic model assessment for insulin resistance (HOMA) was calculated according to the equation: HOMA = ([fasting glucose, mmol/l] × [fasting insulin, μIU/ml])/22,5.

3.3. Flow cytometry

Peripheral blood mononuclear cells (PBMC) were isolated from EDTA-treated blood by centrifuging with Histopaque 1077 (Sigma-Aldrich, USA). PBMC were stained using the following combination of monoclonal antibodies conjugated with the corresponding fluorochromes: anti-CD4-phycoerythrin (PE), anti-CD4-fluorescein isothiocyanate (FITC), and anti-HLA-DR-allophycocyanin (APC) (all reagents: "BD Pharmingen", USA). Cells were analyzed with "FACSCalibur flow cytometer (BD, USA). The percentages of classical CD14++CD16^{lo}, intermediate CD14++CD16^{hi}, and non-classical CD14+CD16^{hi} monocytes were evaluated.

3.4. Imaging flow cytometry

In randomly selected patients, imaging flow cytometry was used to verify the borders for identification of monocyte subpopulations during the conventional flow cytometric analysis (Fig. 1) and to evaluate expression of scavenger receptor CD163. For this purpose, cells were prepared as previously described [3] with anti-CD163-PerCP-Cy5.5 added to the panel of fluorochromes. Cell concentration was adjusted up to 2×10^7 cells/mL. Compensation controls were prepared as cells stained with a single fluorochrome from the panel. Images of the cells were acquired using "Amnis FlowSight" instrument (MERCK, Millipore, USA). The compensation controls were used to create a compensation matrix, which served as a basis for the creation of compensated image files. Cells not in focus were excluded from the further evaluation by gradient RMS feature on the brightfield images. Cell aggregates were excluded using scatter plot Area vs. Aspect ratio (ratio of the shortest axis of the event to the longest axis) on the brightfield images. Only cells with an aspect ratio above 0.6 and medium area were selected. Gates were set on classical CD14++CD16^{lo}, intermediate CD14++CD16^{hi}, and non-classical CD14+CD16^{hi} monocytes using images of the cells. Mean fluorescence intensity (MFI) of CD163 and the counts of cells expressing CD163 were assessed in each population of the monocytes Q3 (see Fig. 2).

3.5. Multiplex analysis

We have performed an extended multiplex analysis of the cytokines, chemokines, and cardio biomarkers in serum and 24-hour PBMC culture medium in 12 randomly selected patients. PBMC were prepared and were either left intact or were stimulated with 10 ug/mL lipopolysaccharide (LPS) for 24 hours. Supernatants were collected and stored at −40° C until the final analysis. Key cardiac markers (BNP, CK-MB, CXCL16, Endocan-1, FABP3, FABP4, LIGHT, CXCL6, NT proBNP, Oncostatin, Placental Growth Factor, and Troponin I), cytokines and chemokines (EGF, VEGF, Eotaxin, FGF-2, Fractalkine, G-CSF, GM-CSF, GRO, IFNα2, IFNγ, IL-1α, IL-1β, IL-1Ra, IL-2, IL-3, IL-4, IL-5, IL-6, IL-7, IL-8/CXCL8, IL-9, IL-10, IL-12 (p40), IL-12 (p70), IL-13, IL-15, IL-17A/CTLA8, IP-10/CXCL10, MCP-1/CCL2, MDC/CCL22, MIP-1α/CCL3, MIP-

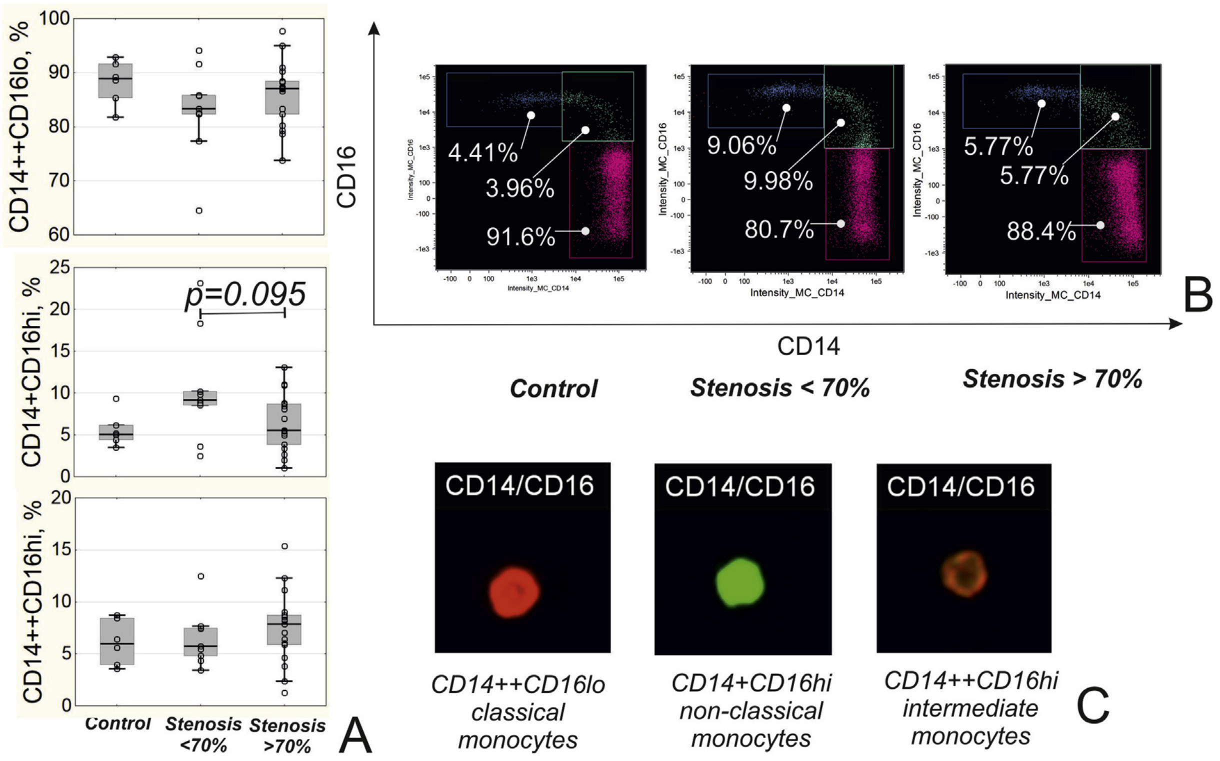

Fig. 1. Subpopulations of monocytes in patients depending on the presence of coronary stenosis. A) Level of different populations of monocytes in patients. B) Representative dot plots of different monocytes subpopulations. C) Representative images of classical (CD14++CD16lo), non-classical (CD14+CD16hi) and intermediate (CD14++CD16hi) monocytes obtained during the imaging flow cytometry.

1β/CCL4, TNFα, and TNFβ/Lymphotoxin-A) were detected by Multiplex Instrument FLEXMAP 3D (Luminex Corporation) using MILLIPLEX map Human Cytokine/Chemokine Panel 1, Human CVD Panel 1, and MILLIPLEX Analyst 5.1 software (Merck KGaA, Milliplex, Darmstadt, Germany). A total of 34 parameters were evaluated. Concentration of cardiac markers, FGF-2, Fractalkine, GRO, IL-13, CD40L, IL-6, MCP-1, MIP-1α, and MIP-1β were analyzed only in serum.

3.6. Statistical analysis

Analysis was performed using Statistica 10 software (StatSoft Inc., USA). The Mann-Whitney U-test was used to estimate the significance of differences between groups. Spearman's rank correlation coefficient (r) was used to estimate relationships between the variables. A value of $p < 0.05$ was considered statistically significant.

4. Results

Patients with coronary stenosis >70% were characterized by a higher duration of AH and DM2 (in diabetic patients), higher values of BMI and waist circumference, and more severe hyperglycemia compared to patients without coronary stenosis or with stenosis <70% (Table 1). The level of hsCRP was elevated in this group (Table 1). Lipid profiles were assessed in patients with and without IHD. Data showed that patients with coronary stenosis ≥70% had lower levels of HDL-cholesterol despite the lower levels of total cholesterol compared to control group and patients with stenosis <70%, which may be due to successful statin therapy (Table 2).

Analysis of monocytes' subpopulation revealed that patients with stenosis <70% tended to have the higher frequency of non-classical CD14+CD16^{hi} monocytes compared to patients with coronary artery stenosis >70% (Fig. 1).

Results of the multiplex analysis showed that patients with stenosis >70% were characterized by the lower levels of IL-6 in serum and the higher levels of spontaneous IL-7 PBMC secretion and LPS-stimulated PBMC secretion of granulocyte colony-stimulating factor (G-CSF) compared to patients with coronary stenosis <70% (Fig. 3). We did not find any significant changes in the concentration of cardiac markers between the patients with and without 70% stenosis, even though patients with stenosis >70%

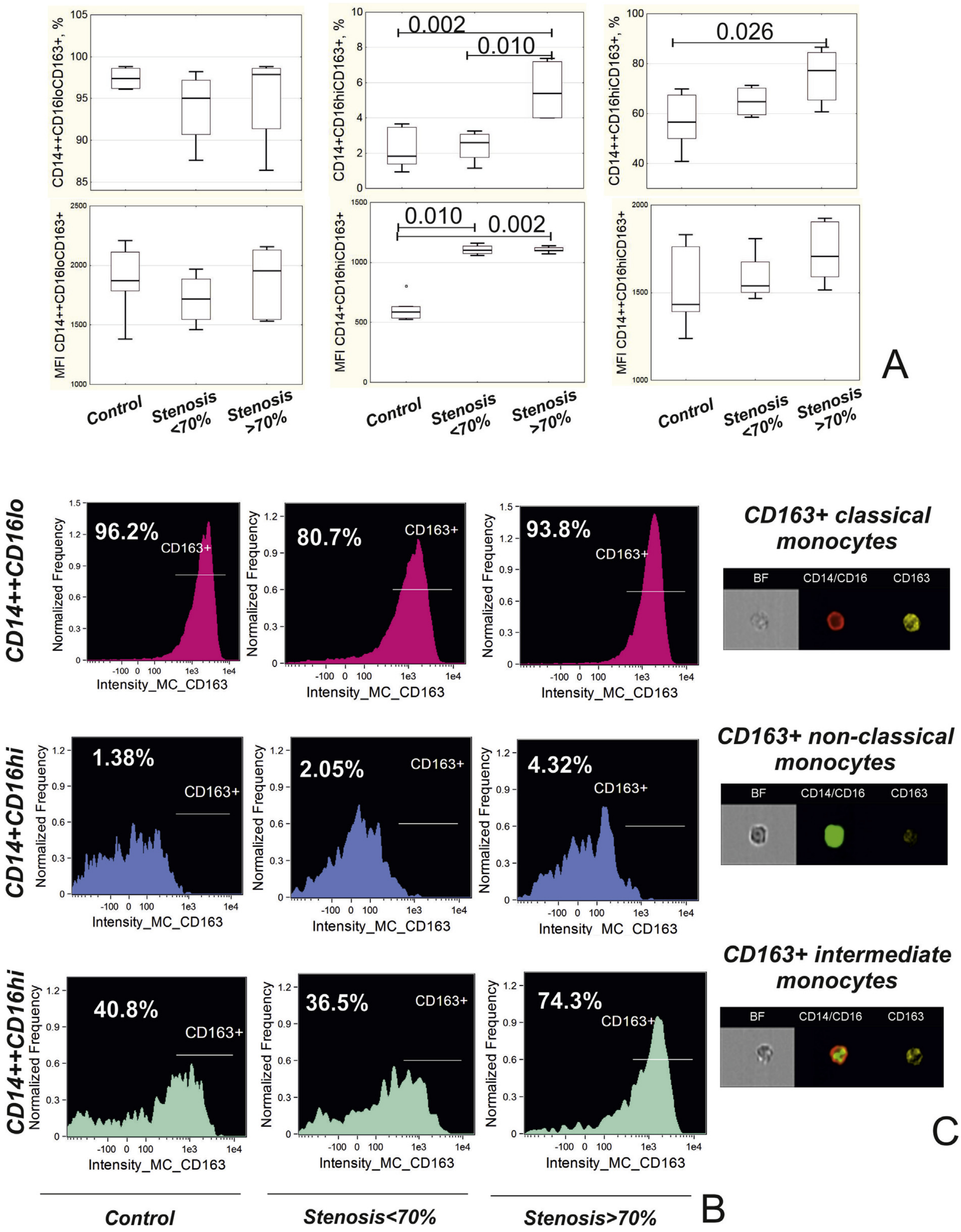

Fig. 2. Expression of CD163 on monocytes in patients depending on the presence of coronary stenosis. A) Frequency and mean fluorescence intensity (MFI) of CD163+ in the subpopulations of classical (CD14++CD16lo), non-classical (CD14+CD16hi) and intermediate (CD14++CD16hi) monocytes. B) Representative histograms of monocytes describing expression of CD163 in different monocyte subpopulations in patient depending on the presence of stenosis. C) Images of CD163+ monocytes obtained during imaging flow cytometry.

Table 2. Lipid profiles in patients with coronary artery disease and in control group (Me (Q1; Q3)).

Parameter	Control group (no IHD) (n = 6)	Patients with coronary stenosis <70% (n = 9)	Patients with coronary stenosis ≥70% (n = 17)	p
Total cholesterol, mM	5.03 (3.92; 6.47)	5.02 (4.44; 5.91)	3.47 (3.10; 4.70)	$p_{1-2} = 0.776$<br>**$p_{1-3} = 0.039$**<br>$p_{2-3} = 0.067$
Triglycerides, mM	1.75 (1.17; 2.31)	1.38 (1.20; 2.74)	1.40 (0.97; 1.63)	$p_{1-2} = 1.000$<br>$p_{1-3} = 0.227$<br>$p_{2-3} = 0.426$
HDL-cholesterol, mM	1.35 (1.25; 1.39)	1.15 (1.10; 1.34)	0.98 (0.78; 1.18)	$p_{1-2} = 0.529$<br>**$p_{1-3} = 0.026$**<br>$p_{2-3} = 0.072$
LDL-cholesterol, mM	3.07 (2.15; 4.15)	3.01 (2.85; 3.23)	1.91 (1.25; 2.88)	$p_{1-2} = 0.689$<br>$p_{1-3} = 0.051$<br>$p_{2-3} = 0.096$
Non-HDL-cholesterol, mM	3.68 (2.95; 5.08)	4.17 (3.40; 4.79)	2.88 (2.10; 4.17)	$p_{1-2} = 0.864$<br>$p_{1-3} = 0.156$<br>$p_{2-3} = 0.212$
TG/HDL-C	1.26 (0.89; 1.58)	1.15 (1.03; 3.04)	1.46 (0.97; 1.80)	$p_{1-2} = 0.954$<br>$p_{1-3} = 0.780$<br>$p_{2-3} = 0.926$
Atherogenic index	2.92 (2.65; 3.66)	3.79 (2.23; 4.25)	2.58 (2.14; 3.90)	$p_{1-2} = 0.689$<br>$p_{1-3} = 0.546$<br>$p_{2-3} = 0.516$
Statins, n (%)	0	6 (66.7%)	15 (88.2%)	$p_{2-3} = 0.208$

HDL-cholesterol – high density cholesterol; LDL-cholesterol – low-density cholesterol; TG- triglycerides.

tended to have higher serum concentration of FABP4 (a marker protein of adipocytes), which probably may be explained by the elevated anthropometric markers of adiposity in this group.

Correlation analysis revealed that the subpopulations of monocytes were associated with different clinical parameters in patients and in control individuals. In control group, non-classical and intermediate monocytes were directly correlated to the ratio TG/HDL-C, whereas the frequency of classical monocytes was reversely correlated with this lipid profile parameter (Fig. 4). In patients with stenosis <70%, the frequency of classical monocytes was positively correlated and frequency of non-classical monocytes was negatively related to the value of the Gensini score (Fig. 4). We also found a direct association between the frequency of intermediate monocytes and waist circumference in this group of patients with stenosis <70% (Fig. 3). In patients with stenosis >70%, there was a direct correlation between the level of hsCRP and the frequency of intermediate CD14++CD16^{hi} monocytes (Fig. 4).

In total group of patients, direct correlations were observed between the concentration of granulocyte/macrophage colony-stimulating factor (GM-CSF) in PBMC culture medium after 24-hour LPS stimulation and GS as well as the frequency of classical CD14++CD16^{lo} monocytes, whereas the frequency of non-classical CD14+CD16^{hi} monocytes was inversely associated with the LPS-stimulated production of GM-CSF by PBMC (Fig. 5). The frequency of non-classical monocytes positively correlated with the serum concentration of CXCL16, whose role in atherosclerosis remains controversial. The frequency of classical monocytes positively correlated with the LPS-stimulated level of TNF-α in 24-hour PBMC culture media (Fig. 5).

5. Discussion

In our work, we showed that the absence of severe stenosis in patients with stable IHD was associated with the increased frequency of non-classical CD14+CD16^{hi} monocytes, inversely associated with the values of GS in this group. Our study was the first one investigating the cohort of IHD patients with the method of imaging flow cytometry to verify the regions during gating of monocyte subpopulations. It is an important point because the subpopulations of classical, non-classical, and intermediate monocytes are indiscrete. An incorrect gating may lead to false results and wrong conclusions.

Currently there is no consensus on the role of various subpopulations of monocytes, non-classical monocytes in particular, in the pathogenesis of atherosclerosis. Some articles emphasize the inflammatory potential of the non-classical CD14+CD16^{hi} monocytes [5, 8, 9]. Others regard

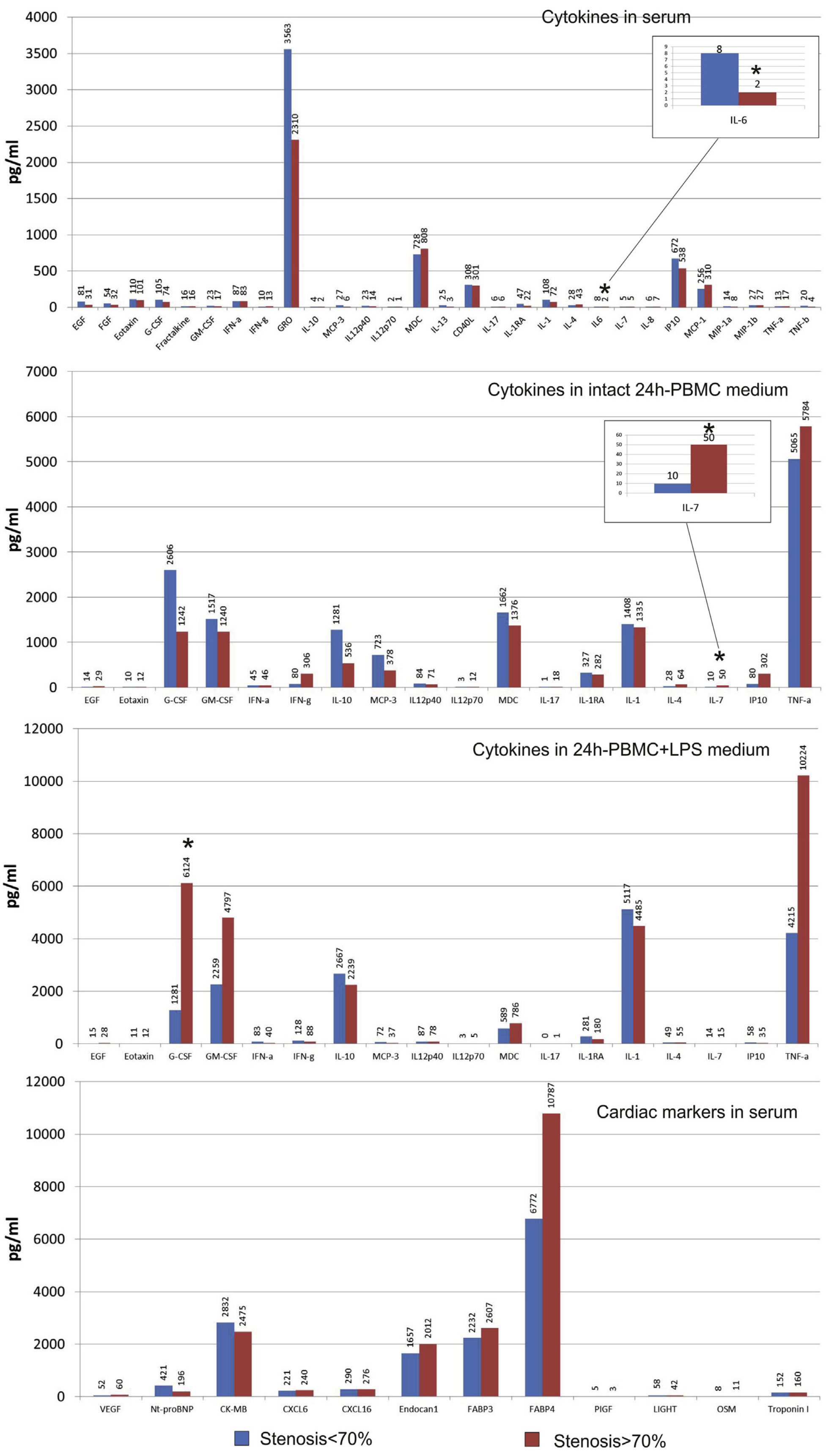

Fig. 3. Concentration of biomarkers in serum and culture medium in patients with IHD.

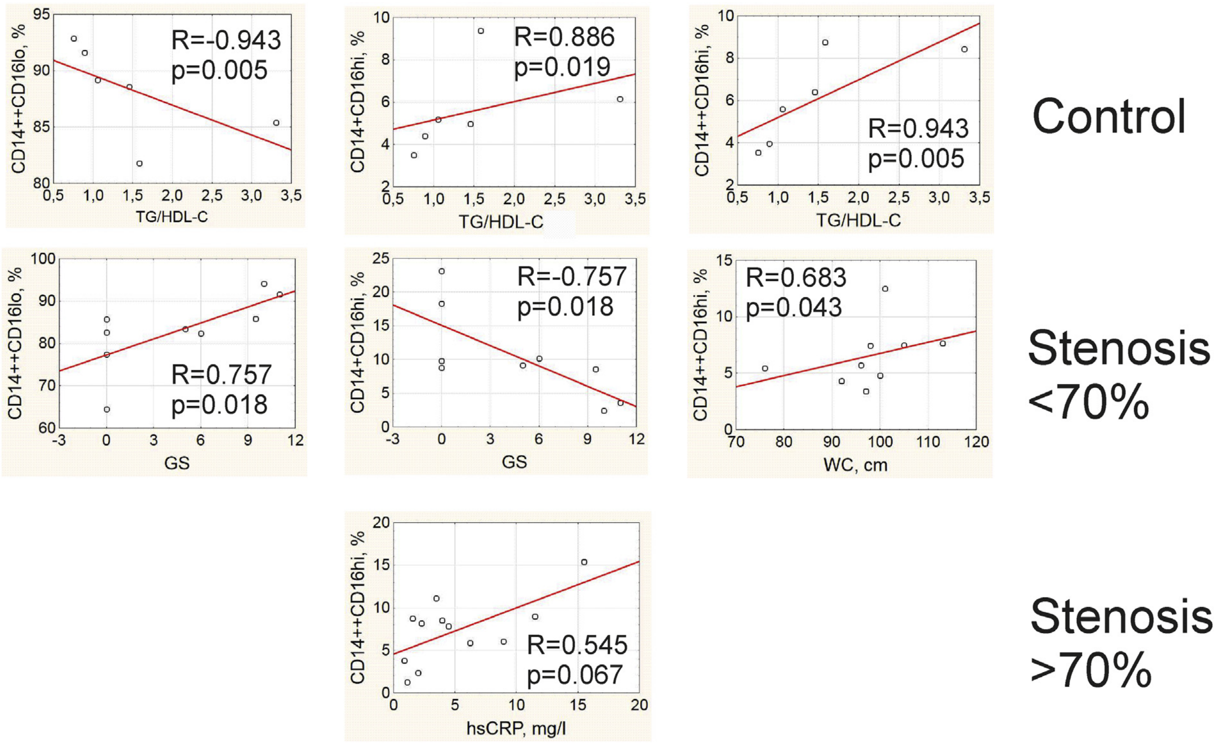

Fig. 4. Correlations between frequency of monocyte subpopulations and clinical parameters.

them rather as a patrolling monocyte subpopulation with primary function to remove debris from the endothelium, as well as oxidized LDL (oxLDL) and apoptic cells [14].

On the one hand, obtained results prompt to suspect non-classical monocytes in patients without stenosis and stenosis <70% to take part in the regulation of the development of inflammation. Indeed, the serum level of hsCRP was lower in group with stenosis <70% in the presence ofLPS-stimulated G-CSF secretion and inverse relationships with LPS-stimulated GM-CSF secretion. We found a direct association between CD14+CD16^{hi} monocytes and concentration of CXCL16, which

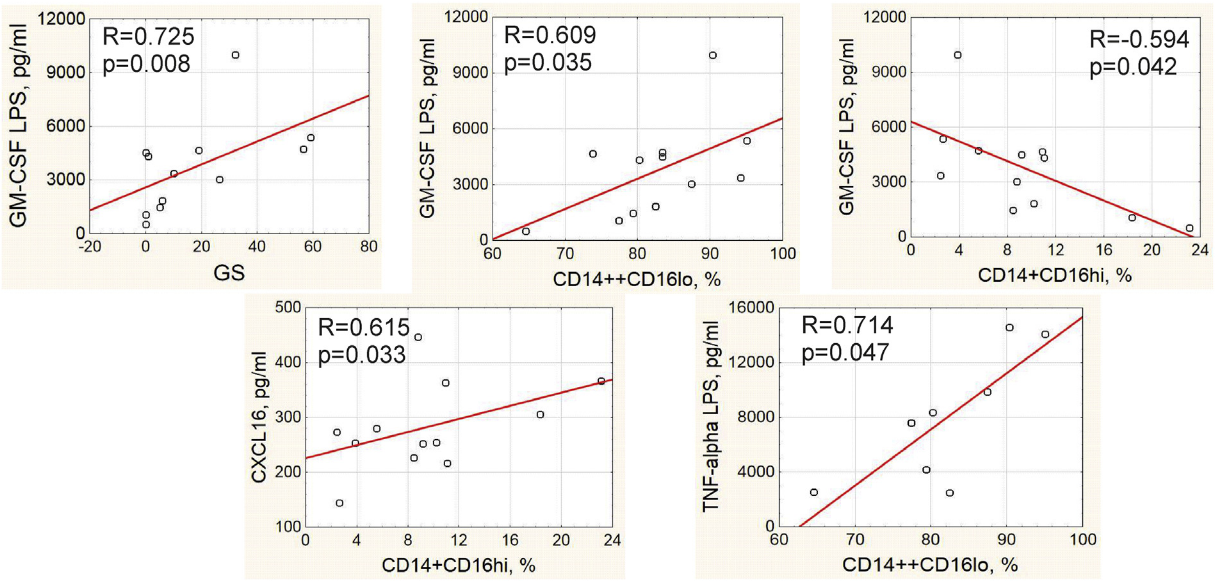

Fig. 5. Correlations between cytokines, monocyte subpopulations and clinical parameters in total group of IHD patients.

goes in accordance with their controversial role in atherosclerosis. The CXCL16/CXCR16 axis is crucially involved in the development of cardiovascular disorders [15]. CXCL16 is expressed by the endothelial cells, smooth muscle cells, macrophages, platelets, and dendritic cells. Being cleaved via proteolysis by ADAM-10 and ADAM-17, it may be found in serum in its soluble form serving as a chemoattractant for the immune cells and recruiting them into the vascular wall [16, 17]. However, CXCL16 exerts certain atheroprotective effects: CXCL16 knockout mice develop bigger aortic plaques compared to wild-type animals. One possible explanation for this controversy may be time-dependent effects of CXCL16 at different stages of atherogenesis [17]. There are also data that CD16+ monocytes are characterized by an increased expression of CXCL16 compared to CD16- monocytes [18]. Even more so, Slan+ (6-sulfo LacNac residue on an O-linked carbohydrate moiety of PSGL-1) non-classical monocytes could actively migrate in the direction of CXCL16 playing atheroprotective role [19].

On the other hand, we suppose that the higher levels of CD14+CD16^{hi} monocytes cannot be regarded as a potential biomarker of more favorable outcome in IHD patients. Development of IHD when stenosis is absent or not severe may be accompanied by the presence of epicardial coronary spasm or endothelial dysfunction [20, 21]. Thus, higher levels of CD14+CD16^{hi} monocytes may be rather a marker of endothelial dysfunction in patients without severe coronary stenosis. Urbanski K. et al. (2017) demonstrated a direct link between the frequency of non-classical monocytes and the potential of arteries to produce reactive oxygen species [22]. Small capillaries were shown to be enriched with non-classical monocytes where they regulate the scavenging of the necrotic endothelial cells [23].

Lipid profile in our study and TG/HDL-C ratio, in particular, was shown to be linked to the numbers of monocyte subpopulations only in patients without IHD. The absence of links between these parameters in both groups of patients with IHD may be partially explained by statin intake by the majority of IHD patients, which could have modified lipid metabolism. Statin intake may also explain discrepancy between data of lipid profile and monocyte subpopulations. According to our results, patients with stenosis <70% had poorer control of lipid profile compared to patients with stenosis ≥70%, even though differences in LDL-C concentration and frequency of statin intake did not reach the level of statistical significance (Table 2). Meanwhile, HDL-C concentration, which is known to be resistant to the effects of statins, was the lowest in patients with severe atherosclerosis. Previous data obtained by other authors showed that HDL-C concentration of is inversely related to the frequency of CD16+ monocytes [24, 25]. In our study, though, it was the ratio of TG/HDL-C that had the strongest link with the monocytes subpopulations. Saja M.F. et al. (2015) recently showed that hypertriglyceridemia induces extravasation of non-classical Ly6C^{lo} monocytes in mice into surrounding tissues [26]. In our case, on the contrary, we observed a direct correlation between TG/HDL-C and circulating CD16+ monocytes. Probably, the effects of lipids on monocyte redistribution may differ between rodents and humans. Understanding of the relationships between dyslipidemia and monocytes functional activity still has many gaps and requires further in-depth studies.

Another factor that may influence the functional activity of monocytes according to our results may be differential expression of haptoglobin-hemoglobin scavenger receptor CD163 depending on the severity of stenosis in patients. Thus, patients with stenosis >70% were characterized by the highest percentage of CD163+ non-classical and intermediate monocytes. Conventionally, CD163 is considered to be a marker of anti-inflammatory M2 macrophages that are thought to be atheroprotective [6, 8]. However, Guo L. et al. recently demonstrated that CD163+ macrophages are able to induce microvascularization and increase permeability and inflammatory cell recruitment into atherosclerotic plaque, which implies an existence of quite an opposite function of these cells as it was originally accepted [27]. CD163 has been recently shown to serve as a scavenger-receptor for the Tumor necrosis factor-like weak inducer of apoptosis (TWEAK). TWEAK has proatherogenic properties, such as induction of proinflammatory cytokines and metalloproteinases production and increase of cellular proliferation and migratory activity [28]. Soluble TWEAK (sTWEAK) decreases while soluble CD163 (sCD163) increases together with the severity of atherosclerosis [29]. We have found no data on the cellular source of sCD163 in patients with atherosclerosis. One cannot exclude that CD16+CD163+ monocytes play a major role in sCD163 production.

The main limitation of our study is a low number of recruited patients, especially in respect of a sample size selected for multiplex analysis. This might have had some impact on the obtained results and can explain limited number of cytokines, chemokines, and cardiac markers that differed

significantly in patients depending on the presence of 70% coronary stenosis.

GM-CSF is a growth factor for cells of the myeloid lineage that has recently been shown to be involved in the pathogenesis of atherosclerosis. Ldlr (−/−) GM−CSF−deficient mice produced less advanced atherosclerosis on western diet, with decreased macrophage and plaque apoptosis, compared to Ldlr (−/−) mice with intact GM-CSF function [30]. For this reason, a strong link revealed in our study between LPS-stimulated production of GM-CSF and GS-values was not surprising. However, the fact that this was accompanied by an indirect correlation with frequency of non-classical $CD14+CD16^{hi}$ monocytes, direct correlation with $CD14{+}{+}CD16^{lo}$ classical monocytes, and the link between $CD14{+}{+}CD16^{lo}$ cells and TNF-α production accentuates even more that classical, but not non-classical monocytes, play the pro-inflammatory role during atherogenesis. This role must take the greatest importance at the severe stages of atherosclerosis, judging by the correlation links between frequency of $CD14{+}{+}CD16^{lo}$ monocytes and hsCRP in patients with stenosis >70%.

There are data that G-CSF prevented the development of atherosclerosis through the mobilization of stem cells from the bone marrow in animal models [31, 32]. In our study, we found that LPS-induced production of G-CSF was already elevated in patients with severer atherosclerosis. Therefore, therapeutic approaches to use G-CSF in patients with stable IHD are unfounded. Our results go in accordance with the work of Z. Hu et al. (2013) who showed that G-CSF aggravates endothelial damage and dyslipidemia, upregulates endothelin-1 expression, and downregulates eNOS in the arterial wall [33]. The prospected study by Katsaros K.M. et al. (2015) demonstrated that serum elevation of G-CSF is an independent predictor of cardiovascular events in stable coronary artery disease [34].

An important immune regulatory cytokine, IL-7, was also elevated in patients with severe atherosclerosis. Of note, we have observed only elevation of the spontaneous production of IL-7 by PBMC. PBMC produced only residual amounts of IL-7 after 24-hour LPS stimulation, which may be the sign of their functional exhaustion. Physiological function of IL-7 is stimulation of T-lymphopoiesis in the thymus and B-cell maturation [35]. However, recent data indicate its involvement in atherogenesis as well. Effects of IL-7 seem to be mediated by the recruitment of monocytes into arterial plaque: IL-7 induces expression of the adhesion molecules and monocyte chemoattractant protein-1 (MCP-1) *in vitro* during incubation with the endothelial cells [36].

The only serum biomarker that differed in patients depending on the presence of 70% stenosis was IL-6, which, on the opposite, was elevated in patients with less severe atherosclerosis. IL-6 plays dualistic role in atherogenesis. On the one hand, it stimulates development of inflammation and is associated with monocytes differentiation towards pro-inflammatory M1-phenotype. On the other hand, IL-6 deficiency leads to the malfunctioning of the LDL-receptor, which, in turn, may aggravate the development of the atherosclerotic lesions [37]. There are data, that IL-6, produced by endothelial cells during hypertension, activates transcriptional factor STAT3 and induces transdifferentiation of the monocytes into CD16+ cells. Reactive oxygen species and NO-deficiency were shown to induce IL-6 production [38]. We have already discussed that an elevation of the non-classical monocytes in the circulation may be associated with the development of endothelial dysfunction in patients with less severe stenosis. Thus, IL-6 may be also involved in this process, playing, at the same time, the regulatory role in the development of atherosclerotic plaques. This hypothesis requires further studies to be undertaken.

6. Conclusions

We conclude that the frequency of non-classical $CD14+CD16^{hi}$ monocytes in patients with stable IHD is indirectly related to the severity of coronary atherosclerosis, whereas classical $CD14{+}{+}CD16^{lo}$ monocytes are directly associated with the Gensini score and inflammatory biomarkers. Expression of CD163 molecules on the intermediate and non-classical monocytes increases in patients with stenosis >70%, which suggests the potential involvement of this molecule in the development of atherosclerosis and associated inflammation. Frequency of monocyte subpopulations is linked to the ratio of TG/HDL-C only in patients without IHD, while monocytes are not related to the lipid profile in IHD patients receiving statin therapy. Overall, our data shed a new light on the involvement of monocytes subpopulations in the pathogenesis of atherosclerosis.

Funding

This work was completed within the framework of the fundamental research № AAAA-A15-115123110026-3. Q5

References

[1] Taleb S. Inflammation in atherosclerosis. Arch Cardiovasc Dis 2016;109:708–15. https://doi.org/10.1016/j.acvd.2016.04.002.

[2] Raggi P, Genest J, Giles JT, Rayner KJ, Dwivedi G, Beanlands RS, et al. Role of inflammation in the pathogenesis of atherosclerosis and therapeutic interventions. Atherosclerosis 2018;276:98–108. https://doi.org/10.1016/j.atherosclerosis.2018.07.014.

[3] Maguire EM, Pearce SWA, Xiao Q. Foam cell formation: A new target for fighting atherosclerosis and cardiovascular disease. Vascul Pharmacol 2019;112:54–71. https://doi.org/10.1016/j.vph.2018.08.002.

[4] Ghattas A, Griffiths HR, Devitt A, Lip GYH, Shantsila E. Monocytes in Coronary Artery Disease and Atherosclerosis: Where Are We Now? J Am College Cardiol 2013;62(17): 1541–51.

[5] Yang J, Zhang L, Yu C, Yang XF, Wang H. Monocyte and macrophage differentiation: circulation inflammatory monocyte as biomarker for inflammatory diseases. Biomark Res 2014;2(1):1. https://doi.org/10.1186/2050-7771-2-1.

[6] Italiani P, Boraschi D. From monocytes to M1/M2 macrophages: phenotypical vs. functional differentiation. Front Immunol 2014:5. https://doi.org/10.3389/fimmu.2014.00514.

[7] Bharat A, McQuattie-Pimentel AC, Budinger GRS. Nonclassical monocytes in tissue injury and cancer. Oncotarget 2017;8(63):106171–2. https://doi.org/10.18632/oncotarget.22584.

[8] Patel VK, Williams H, Li SCH, Fletcher JP, Medbury HJ. Monocyte inflammatory profile is specific for individuals and associated with altered blood lipid levels. Atherosclerosis 2017;263:15–23. https://doi.org/10.1016/j.atherosclerosis.2017.05.026.

[9] Williams H, Cassorla G, Pertsoulis N, Patel V, Vicaretti M, Marmash N, et al. Human classical monocytes display unbalanced M1/M2 phenotype with increased atherosclerotic risk and presence of disease. Int Angiol 2017;36(2):145–55. https://doi.org/10.23736/S0392-9590.16.03661-0.

[10] Rahman K, Vengrenyuk Y, Ramsey SA, Vila NR, Girgis NM, Liu J, et al. Inflammatory Ly6Chi monocytes and their conversion to M2 macrophages drive atherosclerosis regression. J Clin Invest 2017;127(8):2904–15. https://doi.org/10.1172/JCI75005.

[11] Berg KE, Ljungcrantz I, Andersson L, Bryngelson C, Hedblad B, Fredrikson GN, et al. Elevated CD14++CD16- monocytes predict cardiovascular events. Circ Cardiovasc Genet 2012;5(1):122–31. https://doi.org/10.1161/CIRCGENETICS.111.960385.

[12] Lacy M, Atzler D, Liu R, de Winther M, Weber C, Lutgens E. Interactions between dyslipidemia and the immune system and their relevance as putative therapeutic targets in atherosclerosis. Pharmacol Ther 2019;193:50–62. https://doi.org/10.1016/j.pharmthera.2018.08.012.

[13] Amato MC, Giordano C. Visceral adiposity index: an indicator of adipose tissue dysfunction. Int J Endocrinol 2014. https://doi.org/10.1155/2014/730827.

[14] Thomas G, Tacke R, Hedrick CC, Hanna RN. Nonclassical patrolling monocyte function in the vasculature. Arterioscler Thromb Vasc Biol 2015;35(6):1306–16. https://doi.org/10.1161/ATVBAHA.114.304650.

[15] Sheikine Y, Sirsjö A. CXCL16/SR-PSOX–a friend or a foe in atherosclerosis? Atherosclerosis 2008;197(2):487–95. https://doi.org/10.1016/j.atherosclerosis.2007.11.034.

[16] Collado A, Marques P, Escudero P, Rius C, Domingo E, Martinez-Hervaś S. Functional role of endothelial CXCL16/CXCR6-platelet–leucocyte axis in angiotensin II-associatedmetabolic disorders. Cardiovasc Res 2018;114:1764–75. https://doi.org/10.1093/cvr/cvy135.

[17] Jovanović I, Zivković M, Djurić T, Popović M, Alavantić D, Stanković A. CXCL16 in Vascular Pathology Research: from Macro Effects to microRNAs. J Atheroscler Thromb 2015; 22(10):1012–24. https://doi.org/10.5551/jat.29942.

[18] Ancuta P, Liu KY, Misra V, Wacleche VS, Gosselin A, Zhou X, et al. Transcriptional profiling reveals developmental relationship and distinct biological functions of CD16+ and CD16- monocyte subsets. BMC Genomics 2009; 10:403. https://doi.org/10.1186/1471-2164-10-403.

[19] Hamers AAJ, Dinh HQ, Thomas GD, Marcovecchio P, Blatchley A, Nakao CS. Human Monocyte Heterogeneity as Revealed by High-Dimensional Mass Cytometry. Arterioscler Thromb Vasc Biol 2019;39:25–36. https://doi.org/10.1161/ATVBAHA.118.311022.

[20] Wittekoek ME, Piek JJ. Non-obstructive cardiovascular disease: a new challenge for invasive cardiology? Neth Heart J 2018;26:1–2. https://doi.org/10.1007/s12471-017-1062-4.

[21] Sara JDS, Prasad M, Zhang M, Lennon RJ, Herrmann J, Lerman LO, et al. High-sensitivity C-reactive protein is an independent marker of abnormal coronary vasoreactivity in patients with non-obstructive coronary artery disease. Am Heart J 2017;190:1–11. https://doi.org/10.1016/j.ahj.2017.02.035.

[22] Urbanski K, Ludew D, Filip G, Filip M, Sagan A, Szczepaniak P, et al. CD14+CD16++ "nonclassical" monocytes are associated with endothelial dysfunction in patients with coronary artery disease. Thromb Haemost 2017;117(5): 971–80. https://doi.org/10.1160/TH16-08-0614.

[23] Carlin LM, Stamatiades EG, Auffray C, Hanna RN, Glover L, Vizcay-Barrena G, et al. Nr4a1-dependent Ly6Clow monocytes monitor endothelial cells and orchestrate their disposal. Cell 2013;153:362. https://doi.org/10.1016/j.cell.2013.03.010.

[24] Huang ZS, Chiang BL. Correlation between serum lipid profiles and the ratio and count of the CD16+ monocyte subset in peripheral blood of apparently healthy adults. J Formos Med Assoc 2002;101(1):11–7.

[25] Grün JL, Manjarrez-Reyna AN, Gómez-Arauz AY, Leon-Cabrera S, Rückert F, Fragoso JM. High-Density Lipoprotein Reduction Differentially Modulates to Classical and Nonclassical Monocyte Subpopulations in Metabolic Syndrome Patients and in LPS-Stimulated Primary Human Monocytes In Vitro. J Immunol Res 2018:2737040. https://doi.org/10.1155/2018/2737040.

[26] Saja MF, Baudino L, Jackson WD, Cook HT, Malik TH, Fossati-Jimack L. Triglyceride-Rich Lipoproteins Modulate the Distribution and Extravasation of Ly6C/Gr1low Monocytes. Cell Rep 2015;12(11):1802–15. https://doi.org/10.1016/j.celrep.2015.08.020.

[27] Guo L, Akahori H, Harari E, Smith SL, Polavarapu R, Karmali V. CD163+ macrophages promote angiogenesis and vascular permeability accompanied by inflammation in atherosclerosis. J Clin Invest 2018;128(3):1106–24. https://doi.org/10.1172/JCI93025.

[28] Moreno JA, Muñoz-García B, Martín-Ventura JL, Madrigal-Matute J, Orbe J, Páramo JA. The CD163-expressing macrophages recognize and internalize TWEAK: Potential consequences in atherosclerosis. Atherosclerosis 2009;207(1): 103–10. https://doi.org/10.1016/j.atherosclerosis.2009.04.033.

[29] Valdivielso JM, Coll B, Martín-Ventura JL, Moreno JA, Egido J, Fernández E, et al. Soluble TWEAK is associated with atherosclerotic burden in patients with chronic kidney disease. J Nephrol 2013;26(6):1105–13. https://doi.org/10.5301/jn.5000245.

[30] Subramanian M, Thorp E, Tabas I. Identification of a non-growth factor role for GM-CSF in advanced atherosclerosis: promotion of macrophage apoptosis and plaque necrosis through IL-23 signaling. Circ Res 2015;116(2):e13–24. https://doi.org/10.1161/CIRCRESAHA.116.304794.

[31] Hasegawa H, Takano H, Ohtsuka M, Ueda K, Niitsuma Y, Qin Y, et al. G-CSF prevents the progression of atherosclerosis and neointimal formation in rabbits. Biochem Biophys Res Commun 2006;344(1):370–6. 26.

[32] Liu M, Liu K, Chen D, Chen H, Sun K, Ju X, et al. The Effect of Granulocyte Colony-Stimulating Factor on the

Progression of Atherosclerosis in Animal Models: A Meta-Analysis. Biomed Res Int 2017:6705363. https://doi.org/10.1155/2017/6705363.

[33] Hu Z, Zhang J, Guan A, Gong H, Yang M, Zhang G, et al. Granulocyte colony-stimulating factor promotes atherosclerosis in high-fat diet rabbits. Int J Mol Sci 2013;14(3):4805–16. https://doi.org/10.3390/ijms14034805.

[34] Katsaros KM, Speidl WS, Demyanets S, Kastl SP, Krychtiuk KA, Wonnerth A. G-CSF Predicts Cardiovascular Events in Patients with Stable Coronary Artery Disease. PLoS ONE 2015;10(11):e0142532. https://doi.org/10.1371/journal.pone.0142532.

[35] Lin J, Zhu Z, Xiao H, Wakefiwld MR, Ding VA, Bai Q, et al. The role of il-7 in immunity and cancer. Anticancer Res 2017;37(3):963–7. https://doi.org/10.21873/anticanres.11405.

[36] Li R, Paul A, Ko KW, Sheldon M, Rich BE, Terashima T, et al. Interleukin-7 induces recruitment of monocytes/macrophages to endothelium. Eur Heart J 2012;33(24):3114–23. https://doi.org/10.1093/eurheartj/ehr245.

[37] Reiss AB, Siegart NM, De Leon J. Interleukin-6 in atherosclerosis: atherogenic or atheroprotective? Clinical Lipidol 2017;12(1):14–23. https://doi.org/10.1080/17584299.2017.1319787.

[38] Loperena R, Van Beusecum JP, Itani HA, Engel N, Laroumanie F, Xiao L, et al. Hypertension and increased endothelial mechanical stretch promote monocyte differentiation and activation: roles of STAT3, interleukin 6 and hydrogen peroxide. Cardiovasc Res 2018;114(11):1547–63. https://doi.org/10.1093/cvr/cvy112.

AUTHOR QUERY FORM

**Journal:** BIOMED<br><br>**Article Number:** 1015	**Please e-mail your responses and any corrections to:**<br><br>**E-mail:** **conventional@tnq.co.in**

Dear Author,

Please check your proof carefully and mark all corrections at the appropriate place in the proof (e.g., by using on-screen annotation in the PDF file) or compile them in a separate list. **It is crucial that you NOT make direct edits to the PDF using the editing tools as doing so could lead us to overlook your desired changes.** Note: if you opt to annotate the file with software other than Adobe Reader then please also highlight the appropriate place in the PDF file. To ensure fast publication of your paper please return your corrections within 48 hours.

For correction or revision of any artwork, please consult conventional@tnq.co.in.

Any queries or remarks that have arisen during the processing of your manuscript are listed below and highlighted by flags in the proof.

**Location in article**	**Query / Remark: Click on the Q link to find the query's location in text**<br>**Please insert your reply or correction at the corresponding line in the proof**
**Q1**	Please provide affiliation for all authors.
**Q2**	Please provide abstract and keywords.
**Q3**	Please note that 'Fig. 2' was not cited in the text. Please check that the citation(s) suggested by the copyeditor are in the appropriate place, and correct if necessary.
**Q4**	Please provide a definition for the significance of bold in the Tables 1 and 2.
**Q5**	Correctly acknowledging the primary funders and grant IDs of your research is important to ensure compliance with funder policies. We could not find any acknowledgement of funding sources in your text. Is this correct?
**Q6**	Please confirm that given names and surnames have been identified correctly and are presented in the desired order and please carefully verify the spelling of all authors' names.
**Q7**	Please check the retained received and accepted date and correct if necessary.

Thank you for your assistance.
